# Gut microbiota, circulating metabolites, and gallstone disease: a Mendelian randomization study

**DOI:** 10.3389/fmicb.2024.1336673

**Published:** 2024-01-25

**Authors:** Xutao Hu, Qiu Binxu, Guang-zhao Shao, Yu Huang, Wei Qiu

**Affiliations:** ^1^Department of Hepatobiliary and Pancreatic Surgery, First Hospital of Jilin University, Changchun, Jilin, China; ^2^Department of Gastric and Colorectal Surgery, General Surgery Center, The First Hospital of Jilin University, Changchun, China

**Keywords:** gut microbiota, circulating metabolites, gallstone disease, Mendelian randomization, omega-3 polyunsaturated fatty acids

## Abstract

**Background:**

The link between Gut microbiota (GM) and Gallstone disease (GSD) is well established, but it is not clear whether there is a causal relationship between the two associations.

**Methods:**

We conducted bidirectional Mendelian randomization (MR) analyses, leveraging aggregated data from the Genome-Wide Association Study (GWAS) of GM and Circulating Metabolites. Our primary objective was to investigate the causal interplay between intestinal flora and GSD. Additionally, we performed mediational analyses, two-step MR, and multivariate MR to uncover the potential mediating effect of circulating metabolites in this relationship.

**Result:**

Our study has revealed a causal relationship between GSD and six distinct bacterial groups. Genetically predicted Class Bacilli (Odds Ratio (OR): 0.901, 95% Confidence Interval (95% CI): 0.825–0.985; *p =* 0.021), Order Lactobacillales (OR: 0.895, 95% CI: 0.816–0.981; *p =* 0.017), and Genus Coprococcus 2 (OR: 0.884, 95% CI: 0.804–0.973; *p =* 0.011) were inversely associated with the risk of GSD. Conversely, the Genus Clostridiumsensustricto1 (OR: 1.158, 95% CI: 1.029–1.303; *p* = 0.015), Genus Coprococcus3 (OR: 1.166, 95% CI: 1.024–1.327; *p* = 0.020), and Genus Peptococcus (OR: 1.070, 95% CI: 1.017–1.125; *p =* 0.009) were positively associated with the risk of GSD. Moreover, our findings suggest that the positive influence of the Genus Peptococcus on GSD may be mediated through Omega-3 polyunsaturated fatty acids (PUFA).

**Conclusion:**

This study reinforces the connection between the gut microbiome and the risk of GSD while also unveiling the mediating role of Omega-3 PUFA in the causal relationship between these factors.

## Introduction

Gallstone disease (GSD) has emerged as one of the most prevalent gastrointestinal disorders in Western countries (Bodmer et al., 2009). In the United States, GSD ranks as the second most common digestive ailment, trailing only gastroesophageal reflux disease (Marschall and Einarsson, 2007). It’s worth noting that nearly 90% of gallstones belong to the cholesterol type (Lammert et al., 2016). Previous epidemiological investigations have pinpointed several risk factors associated with symptomatic GSD, including obesity (Tsai et al., 2006), smoking (Aune et al., 2016), low plasma high-density lipoprotein (HDL) cholesterol (Tsai et al., 2006), and hyperinsulinaemia (Diehl, 2000). Moreover, it is crucial to acknowledge that gallstones not only pose immediate health risks but are also linked to heightened mortality and a greater likelihood of developing major chronic conditions like diabetes, cardiovascular disease, and cancer. These associations likely stem from shared pathways and risk factors.

The human Gut microbiota (GM) is composed of approximately 2,000 diverse bacterial species and plays a pivotal role in maintaining overall health (Caruso et al., 2020). Gallstone formation is primarily influenced by factors such as impaired gallbladder dynamics, abnormal cholesterol metabolism, and irregular bile acid secretion (Wu et al., 2013). In recent years, there has been a growing body of evidence suggesting that imbalances in the GM are pivotal in the development of GSD. Recent studies have even demonstrated that transplanting GM from individuals with gallstones into susceptible mouse strains can induce gallstone formation (Hu et al., 2022). Notably, a direct correlation has been established between bile acid profiles in GSD and the alpha diversity of the GM (Petrov et al., 2020). Furthermore, Takeda and colleagues found that supplementation with clostridial butyric acid not only reduced the incidence of gallstones but also facilitated their dissolution (Takeda et al., 1983). However, considering that the present study is predominantly observational and susceptible to constraints related to sample size and confounding factors, further comprehensive investigation is essential to explore the causal relationship between GM and GSD, as well as to uncover the underlying mechanisms of this association.

Clinical research and animal studies have provided compelling evidence of a connection between the GM and the risk of GSD. This connection may be mediated by its effects on specific blood metabolites, such as trimethylamine N-oxide and short-chain fatty acids (Arpaia et al., 2013; Chen et al., 2019). However, the precise relationship between genetic modifications and circulating metabolites remains inadequately understood. Therefore, our goal is to elucidate these associations and identify potential metabolites that can serve as crucial tools for early diagnosis and clinical therapeutic interventions.

Mendelian randomization (MR) is a data analysis method that leverages single nucleotide polymorphisms (SNPs), which are strongly associated with a phenotype, as instrumental variables (IVs) to investigate causality (Krumsiek et al., 2012). This approach helps circumvent the limitations commonly encountered in traditional observational studies, including issues related to confounding factors and reverse causation. It provides more robust evidence for drawing causal inferences. Consequently, in this study, we conducted MR analyses using the most up-to-date Genome-Wide Association Study (GWAS) dataset for GM, blood metabolites, and GSD. Our aim was to explore the causal relationship between GM and GSD, as well as the potential mediating role of circulating metabolites in this relationship.

## Method

### Data source

The MiBioGen Study furnishes genome-wide association summary data for the human GM (Kurilshikov et al., 2021). This expansive research consortium encompasses a wealth of data from 24 cohorts, collectively involving 18,340 participants hailing from diverse regions, including the United States, Canada, Israel, Korea, Germany, Denmark, the Netherlands, Belgium, Sweden, Finland, and the United Kingdom. The majority of participants in this study have European ancestry, amounting to 13,266 individuals. The microbial composition was assessed by focusing on three distinct variable regions, and conducted a microbiota quantitative trait loci (mbQTL) mapping analysis to pinpoint host genetic variants associated with the abundance of gut bacterial taxa. This meticulous curation process yielded a total of 211 taxonomic units. However, 15 unclassified taxonomic units were excluded from analysis, resulting in a dataset that incorporates 196 bacterial taxonomic units. These units span across 9 phyla, 16 orders, 20 classes, 32 families, and 119 genera, providing a comprehensive foundation for in-depth analysis.

We acquired GWAS data for circulating metabolites from the Nightingale Health Study, which encompassed 12,100 participants of European descent (Ritchie et al., 2023). This extensive dataset comprises a total of 249 circulating metabolites, encompassing lipoproteins, lipids, fatty acids, fatty acid composition, and a wide array of low molecular weight metabolites, such as amino acids, ketone bodies, and glycolytic metabolites. In our study, we included the absolute concentrations of 168 biomarkers and excluded the ratios of 81 biomarkers. Comprehensive GWAS statistics can be accessed by the public through the IEU Open-GWAS project database (Supplementary Table 1) (Elsworth et al., 2020).

Summary statistics for GSD in the GWAS were obtained from the FinnGen Consortium R8 release, with the “cholelithiasis” phenotype corresponding ICD number (ICD-10: K80, ICD-9: 574, ICD-8: 574) being the primary focus of our study. This extensive GWAS dataset included a substantial cohort comprising 32,894 cases and 301,383 controls. To ensure the robustness and reliability of our findings, rigorous adjustments throughout the analysis were applied, accounting for principal components, including gender and age, as well as variations in genotyping batches.

### Selection criteria of IVs

**Figure 1:**
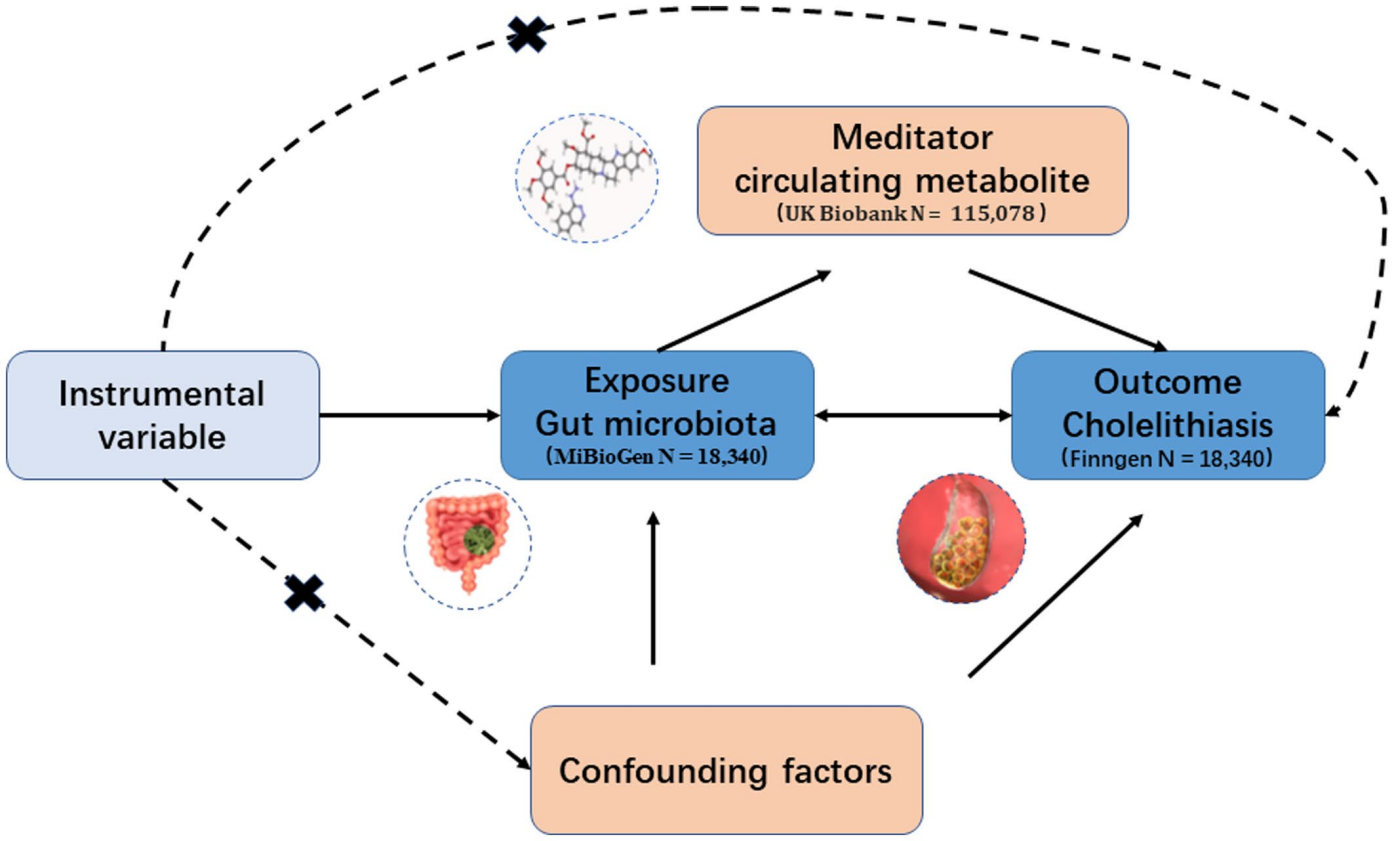
Hypothesis and design of bidirectional and mediated MR analyses. First, bidirectional UVMR was performed to investigate the causal relationship between GM and GSD. Second, 168 blood metabolites (mediators) were selected for subsequent mediation analysis. Finally, a two-step MR analysis was performed to detect potential mediating metabolites.

In this study, we conducted a two-sample MR analysis as a method for causal inference to establish the validity of the causal effect. The validity of the MR analysis relied on three fundamental assumptions: (1) IVs are not linked to any confounding variables; (2) IVs exhibit a strong correlation with the exposure; and (3) IVs influence the outcome solely through the exposure (Figure 1).

Regarding the GM data, we aimed to maintain data reliability and ensure an adequate number of SNPs for exposure analysis. To achieve this, we referred to previous MR studies (Sanna et al., 2019; Liu et al., 2022). We set a genome-wide threshold for exposure-associated SNPs at 1 × 10^−5^. For SNPs associated with GSD and blood metabolites, we applied the standard GWAS thresholds (*p* < 5 × 10^−8^). Additionally, we utilized a chained unbalanced aggregation method (*r*^2^ < 0.001, window size >10,000 kb) to eliminate unwanted SNPs, ensuring their independence (Liu et al., 2023). We selected SNPs with effect allele frequencies >0.01 and excluded SNPs with *F*-statistics <10 to maintain data quality. *F*-statistics were calculated using the formula *F* = Beta^2^/SE^2^.

### Statistical analyses

We used two-sample bivariate univariate MR (UVMR) to assess the causal effect of GM on GSD. We used a variety of analyses, including inverse variance weighting (IVW), MR Egger and weighted median, with IVW as the primary analysis (Lin et al., 2021). To assess the potential impact of directed pleiotropy, we examined the intercept value of the MR-Egger regression. When the regression intercept was non-zero and the *p* < 0.05, we regarded it as a statistically significant indicator of genetic pleiotropy. Heterogeneity was assessed using Cochran’s Q test (Zhang et al., 2023). In the presence of heterogeneity, we first performed a random-effects IVW analysis. All IVW results were corrected for multiple testing using the False Discovery Rate (FDR) method and FDR q-values were provided. We considered results that remained positive after FDR correction as strong causal associations, whereas an FDR *q* > 0.05 was considered a potential causal association with GSD.

We employed a two-step Mendelian randomization (TSMR) method to investigate the potential mediating role of circulating metabolites between GM and GSD (Sanderson, 2021). First, we conducted UVMR to estimate the effect of GM on GSD (α). Subsequently, we utilized UVMR to identify metabolites significantly associated with GSD and adjusted for the genetic effects of GM through multivariate Mendelian randomization (MVMR) to assess the impact of these metabolites on GSD (β2). Lastly, we employed UVMR to evaluate the effect of GM associated with GSD on metabolites (β1). The mediation ratio was calculated as β1*β2/α to quantify the mediating influence of metabolites in the relationship between GM and GSD.

All analyses were conducted in the R Studio environment, utilizing R version 4.3.1. We employed the R packages “TwoSampleMR,” “MendelianRandomisation,” and “MVMR” for the estimation of FDR q-values. The estimation of FDR q-values was accomplished using the R package “p.adjust.”

## Results

### Instrumental variable screening

Following the criteria for IVs selection, we chose a variable number of SNPs, ranging from 4 to 26, as instrumental variables from among 196 GM taxa. For the 168 metabolites, the number of SNPs selected ranged from 7 to 122. In the FinnGen database, 57 SNPs were associated with GSD, as detailed in Supplementary Tables 2–4. Importantly, all instrumental variables exhibited F-statistics greater than 10, signifying the absence of weak instrumental bias in this study.

### Two-sample and bidirectional MR analyses of GM and GSD

**Figure 2:**
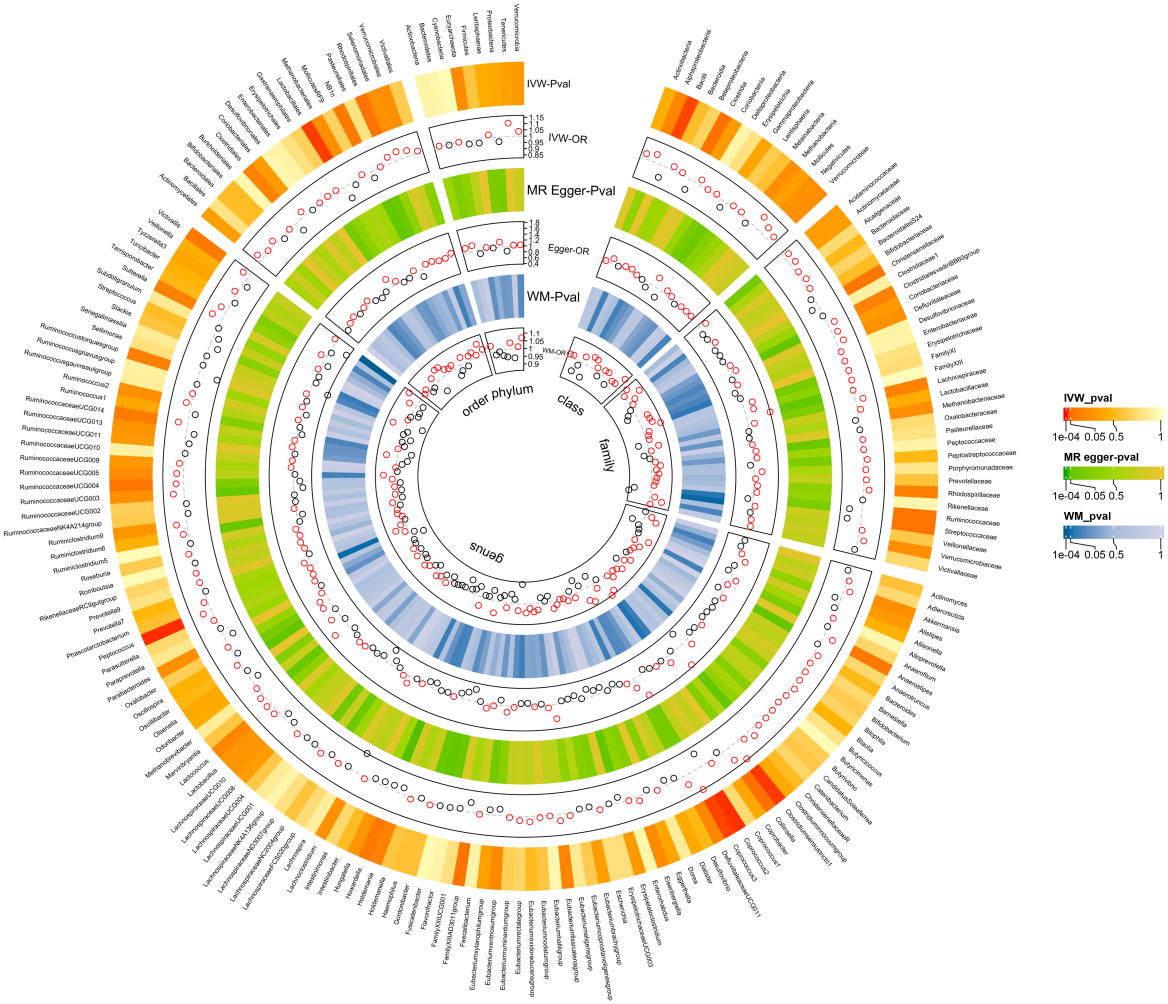
All results of MR analysis and sensitivity analysis between GM and GSD.

**Figure 3:**
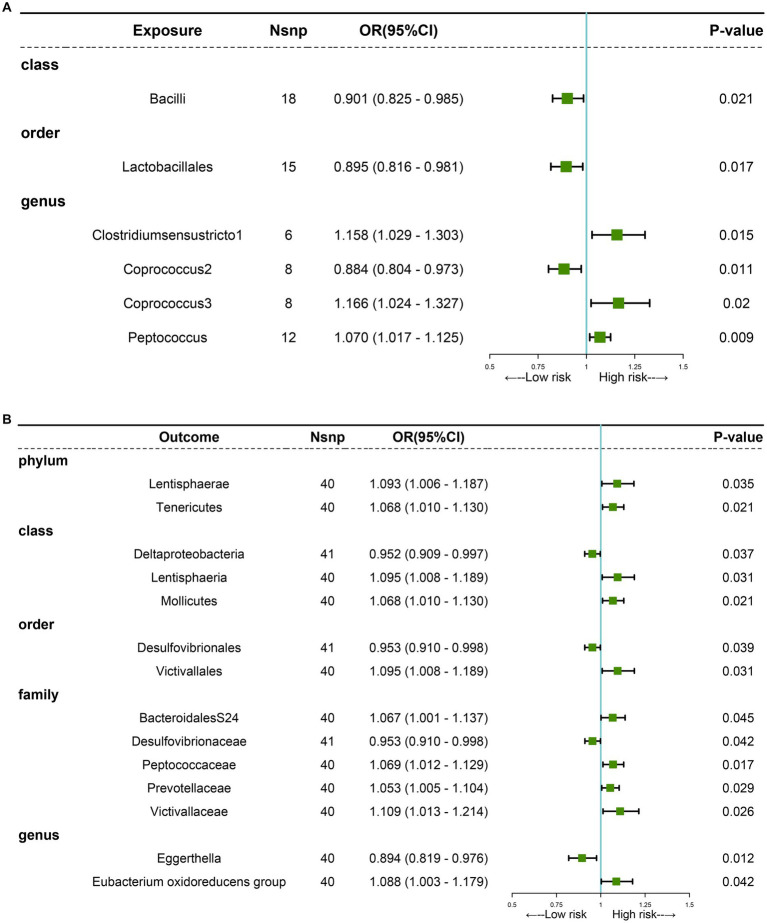
MR analysis showing causality between GM and GSD using the IVW method (*p* < 0.05). **(A)** Causal effect of GM on GSD. **(B)** Causal effect of GSD on GM.

The MR analyses uncovered a causal relationship between six intestinal flora components and GM including one order, one phylum, and three genus (Figures 2, 3A and Supplementary Table 5). Specifically, we observed that the genetically predicted Class Bacilli (Odds Ratio (OR): 0.901, 95% Confidence Interval (95%CI):0.825–0.985; *p =* 0.021), Order Lactobacillales (OR: 0.895, 95%CI:0.816–0.981; *p* = 0.017), and the Genus Coprococcus2 (OR: 0.884, 95%CI:0.804–0.973; *p =* 0.011) were associated with a reduced risk of developing GSD. Conversely, the Genus Clostridiumsensustricto1 (OR: 1.158, 95% CI: 1.029–1.303; *p =* 0.015), the Genus Coprococcus3 (OR: 1.166, 95% CI: 1.024–1.327; *p* = 0.020), and the Genus Peptococcus (OR: 1.070, 95% CI: 1.017–1.125; *p =* 0.009) were linked to an elevated risk of GSD. The MR-Egger test and Cochrane’ *Q* test did not reveal significant horizontal pleiotropy and heterogeneity.

Subsequently, we performed a reverse analysis. Following the onset of GSD, the relative abundance of most prominent taxa increased, including two phylums, two orders, one class, four families, and one genus, while four taxa exhibited significant decreases in relative abundance (Figure 3B and Supplementary Table 6). Particularly, the Genus Eggerthella was most significantly affected by GSD (OR: 0.894, 95% CI: 0.819–0.976; *p =* 0.012).

In MR-Egger and Cochrane’s *Q* tests, we did not detect significant horizontal pleiotropy and heterogeneity. The sensitivity analysis results were generally consistent with the IVW estimates, underscoring the robustness of the association between GM and the risk of GSD. The leave-one-out analysis method did not reveal any interference with the results attributable to a single SNP.

### Mediation analysis of potential blood metabolites

**Figure 4:**
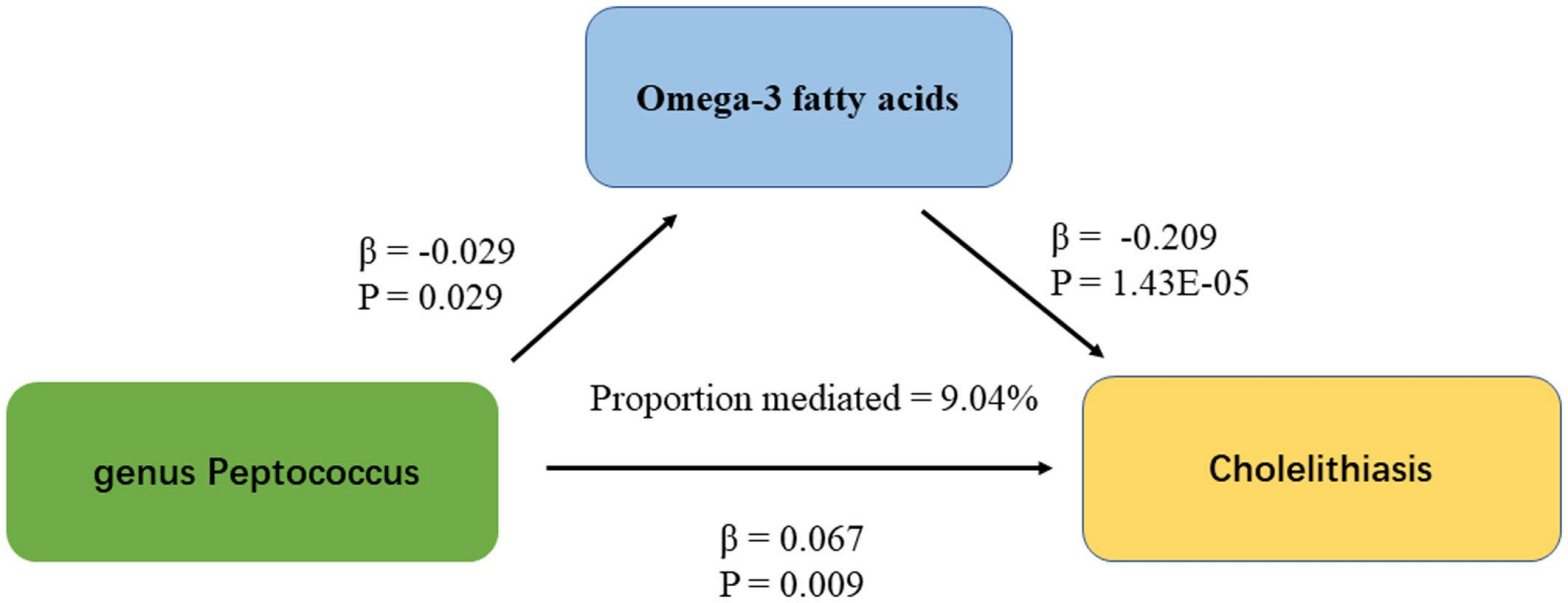
The figure shows the mediation pattern of “GM – blood metabolites – GSD” in a TSMR. β indicates the estimate of the causal effect using an IVW method (*p* < 0.05).

IVW results showed a causal association between 26 plasma metabolites and GSD (Table 1). Specifically, there was a significant association between genetically predicted Omega-3 polyunsaturated fatty acids (PUFA) (OR: 0.823, 95% CI: 0.751–0.902, *p =* 3.43E-05) and Docosahexaenoic acid (OR 0.819,95% CI 0.743–0.903, *p =* 6.10E-05). MR-Egger test did not show significant horizontal pleiotropy (Supplementary Table 7). Among the six intestinal flora causally associated with GSD, we found genetically predicted Genus Peptococcus with Omega-3 PUFA (OR 0.972, 95% CI: 0.947–0.997, *p =* 0.029, Supplementary Table 8). The findings did not reveal evidence of heterogeneity and horizontal pleiotropy. We further used MVMR to assess the independent effect of Omega-3 PUFA on GSD. The effect of Omega-3 PUFA was found to remain significant after adjusting for GM (OR 0.811, 95% CI 0.811–0.892, *p =* 1.43E-05, Table 2). The results of the study showed that Genus Peptococcus mediated 9.04% of the total effect by reducing the concentration of Omega-3 fatty acids on the adverse effects of GSD (Figure 4).

**Table 1:** The effects of the above 168 metabolites on GSD using an IVW method (*p* < 0.05).

Exposure	Nsnp	OR(95%CI)	*p*-value	*Q* statistic	P-heterogeneity	Egger intercept	P-intercept
Omega-3 PUFA	4	0.661 (0.494–0.884)	5.27E-03	1.40	7.06E-01	−0.003	9.07E-01
Docosahexaenoic acid	24	1.372 (1.085–1.736)	8.29E-03	181.95	1.09E-26	−0.021	2.35E-02
Acetoacetate	50	0.817 (0.682–0.978)	2.77E-02	598.52	1.43E-95	0.006	4.99E-01
Degree of unsaturation	35	0.819 (0.743–0.903)	6.10E-05	163.34	7.89E-19	0.001	9.25E-01
Albumin	34	0.939 (0.894–0.987)	1.35E-02	91.16	2.37E-07	0.005	1.31E-01
Total esterified cholesterol	40	0.803 (0.674–0.957)	1.44E-02	299.45	6.80E-42	0.021	8.55E-03
Sphingomyelins	11	1.551 (1.063–2.264)	2.28E-02	75.59	3.65E-12	0.003	9.14E-01
Total concentration of lipoprotein particles	54	0.799 (0.644–0.991)	4.15E-02	877.60	8.69E-150	−0.006	5.74E-01
Glycine	53	0.800 (0.641–0.997)	4.75E-02	878.11	1.65E-150	−0.007	5.50E-01
Glycoprotein acetyls	52	0.796 (0.644–0.983)	3.43E-02	836.28	1.45E-142	−0.006	5.81E-01
Triglycerides in large LDL	47	0.867 (0.764–0.984)	2.73E-02	407.01	2.54E-59	0.005	4.40E-01
Histidine	44	0.904 (0.824–0.992)	3.35E-02	234.55	3.35E-28	−0.001	8.36E-01
Phosphoglycerides	48	0.771 (0.597–0.996)	4.66E-02	1052.35	1.00E-189	−0.006	6.15E-01
Average diameter for LDL particles	52	0.864 (0.762–0.979)	2.19E-02	391.78	4.46E-54	0.009	1.38E-01
Triglycerides in IDL	30	0.800 (0.657–0.975)	2.71E-02	168.31	1.42E-21	0.000	9.68E-01
Total cholines	55	0.864 (0.752–0.994)	4.05E-02	408.54	6.40E-56	0.001	8.65E-01
Triglycerides in large HDL	41	0.772 (0.598–0.996)	4.65E-02	809.86	4.14E-144	−0.015	2.19E-01
Free cholesterol in IDL	39	0.823 (0.751–0.902)	3.43E-05	224.00	3.27E-28	−0.004	4.97E-01
Total cholesterol	49	0.820 (0.688–0.977)	2.62E-02	578.66	3.80E-92	0.007	4.52E-01
Phospholipids in medium HDL	53	0.842 (0.738–0.961)	1.06E-02	328.86	7.39E-42	0.010	1.38E-01
Cholesterol in IDL	51	0.743 (0.561–0.983)	3.78E-02	1271.58	2.42E-233	−0.001	9.56E-01
Total free cholesterol	53	0.723 (0.564–0.927)	1.04E-02	1040.00	7.91E-184	−0.009	4.93E-01
Cholesteryl esters in medium VLDL	47	0.743 (0.557–0.992)	4.39E-02	1258.62	1.71E-233	−0.004	7.73E-01
Free cholesterol in large LDL	53	0.830 (0.718–0.959)	1.14E-02	359.80	1.32E-47	0.004	5.30E-01
Cholesteryl esters in IDL	52	0.773 (0.599–0.998)	4.84E-02	1177.32	5.48E-213	−0.001	9.43E-01
Total pospholipids in lipoprotein particles	35	0.857 (0.767–0.958)	6.70E-03	245.99	5.77E-34	0.005	4.17E-01

GSD, Gallstone disease; IVW, Inverse variance weighting; OR, Odd ratio; 95% CI, 95% Confidence intervals; LDL, Low density lipoprotein; HDL, High density lipoprotein; IDL, Intermediate density lipoprotein; VLDL, Very low-density lipoprotein.

**Table 2:** Multivariate MR results of the effect of circulating metabolites on GSD after adjusting for GM.

Exposure	Method	OR	95%CI	*p*-value	*Q* statistic	P-heterogeneity	Egger intercept	P-intercept
Omega-3 PUFA	MV-IVW	0.811	0.738–0.892	1.43E-05	201.7701	1.83E-23		
Omega-3 PUFA	MV-EGGER	0.814	0.740–0.895	2.12E-05	197.4758	4.60E-23	0.005	0.357

GSD, Gallstone disease; IVW, Inverse variance weighting; OR, Odd ratio, 95% CI, 95% confidence intervals and *p*-values were calculated for the corresponding MR analysis methods. Heterogeneity tests in the IVW method were performed using Cochran’s *Q* statistic. *p* < 0.05 was considered significant.

## Discussion

To our best understanding, our research stands as the pioneering utilization of MR to probe into the causal link between GM and GSD, while simultaneously exploring the plausible mediating function of circulating metabolites within this connection. In this investigation, we identified six distinct taxa with an established causative connection to GSD. Furthermore, we utilized TSMR techniques to explore the potential intermediary role of circulating metabolites in the causative link between gut flora and GSD. Notably, our discovery of a positive driving influence of Genus Peptococcus on the susceptibility to GSD may be attributed to its potential mediation of reduced Omega-3 PUFA concentrations.

Cholesterol stones constitute more than 80% of GSD cases, and disturbances in bile acid metabolism play a significant role in the pathogenesis of cholesterol GSD (Lammert et al., 2016). The intestinal microbiota can further impact the formation of GSD through its involvement in various processes, including influencing bile acid composition, participating in the cleavage of exogenous aryl rings, deconjugating bile acid complexes via hydrolytic enzymes, regulating free bile acids, and modulating the circulation of bile acids within the liver and intestines (Molinero et al., 2019).

In our study, we identified three GM components that appeared to offer a nominal protective effect against GSD. These included Class Bacilli, Order Lactobacillales, and Genus Coprococcus2. Previous research has revealed that Order Lactobacillales and Genus Coprococcus2 produce bile salt hydrolase, an enzyme that facilitates the conversion of primary bile acids into secondary bile acids (Molinero et al., 2019). This conversion process leads to an increase in free bile acids within the intestinal lumen, reducing their reabsorption by the intestine and consequently increasing the excretion of bile acids in the feces (Islam et al., 2011). The increased excretion of bile acids serves to reduce the enterohepatic circulation of these compounds. To sustain the requisite levels of conjugated bile acids necessary for the enterohepatic circulation, the liver intensifies the conversion of cholesterol into bile acids, which, in turn, leads to a reduction in serum cholesterol levels (Wang et al., 2012). Recent studies have found that Order Lactobacillales may promote farnesoid X receptor (FXR) activation by lowering intestinal tauro-α-muricholic acid (T-α-MCA) and tauro-β-MCA (T-β-MCA) concentrations, reducing bile acids (BAs) and cholesterol synthesis in the liver and thereby reducing the incidence of GSD (Ye et al., 2022). Furthermore, Genus Coprococcus2 plays a role in the production of short-chain fatty acids (SCFA) within the intestine (Nagpal et al., 2018). SCFAs are known to play a significant role in maintaining intestinal homeostasis and providing resistance against inflammation (Yao et al., 2022). Notably, butyrate, a product of SCFA metabolism, has been demonstrated to influence blood cholesterol levels and bile acid metabolism (Ye et al., 2021).

In our research, the Genus Clostridiumsensustricto1 displayed a positive association with the risk of GSD. Several earlier investigations have documented an association between Clostridium and GSD (Grigor'eva and Romanova, 2020; Li et al., 2023). These findings indicate that Clostridiumsensustricto1 could potentially be considered a contributing factor to GSD. Genus Clostridiumsensustricto1 is known to produce β-glucuronidase (Trotman, 1991), an enzyme that leads to the deconjugation of bilirubin diglucosidic acid. This deconjugation can result in the precipitation of bilirubin calcium, which, in turn, contributes to the formation of biliary stones (Cetta, 1991; Leung et al., 2001). Regarding Genus Peptococcus, there have been limited studies on its relationship with GSD. One previous study has suggested that an increased abundance of Genus Peptococcus may be associated with elevated lipid and cholesterol levels in the liver and plasma (Kwek et al., 2022). However, further research is needed to explore this relationship in greater depth.

Our MR study delivers compelling genetic evidence that firmly establishes a causal relationship between two unsaturated fatty acids and the risk of GSD. Elevated levels of Omega-3 PUFA and Docosahexaenoic acid are correlated with a reduced risk of GSD. Nevertheless, it is important to note that existing epidemiological and clinical studies exploring the association between PUFA and the risk of GSD have yielded conflicting and limited results. In a population-based prospective cohort study, it was found that the consumption of PUFA and monounsaturated fats (MUFA) seemed to be linked to a decreased risk of GSD in men (Tsai et al., 2004). However, it’s important to acknowledge that another study reported no significant associations between the type or subtype of total dietary fat and the formation of biliary stones or sludge (Mathew and Ko, 2015). These conflicting results from epidemiological studies may have limitations in establishing a causal relationship between GM and GSD.

Through our mediation analyses, we have presented genetic evidence supporting the connection between GM and blood metabolites. As far as our knowledge extends, no previous studies have established a connection between Peptococcus and circulating Omega-3 PUFA. Previous experiments have provided evidence that the metabolism of GM can influence the absorption, bioavailability, and biotransformation of PUFA. For instance, previous research has demonstrated that GM can lower Omega-3 PUFA levels by promoting the conversion of PUFA precursors like alpha-linolenic acid (ALA) and linoleic acid (LA) into conjugated linoleic acid (CLA) and hydrogenating conjugated alpha-linolenic acid (CALA) into saturated fatty acids, such as stearic acid (Jenkins et al., 2008; Shama and Liu, 2020). Xu H. et al. research revealed that specific gut bacteria can increase the levels of omega-3 oxysphospholipids, derived from omega-3 PUFA, with potential benefits for both systemic and gut health (Xu et al., 2022). Another earlier study has provided insights into how the GM produces enzymes involved in pathways related to fatty acid metabolism, thus enabling the conversion of PUFA in the gastrointestinal tract (Kishino et al., 2013). These findings, in conjunction with our study results, establish a foundation for a causal relationship between GM and the regulation of PUFA metabolism.

Our study boasts several strengths. To the best of our knowledge, it is the first investigation to employ MR analysis to explore the connections between the gut microbiome, circulating metabolites, and GSD within the general population. Furthermore, our study furnishes genetic evidence for a plausible mechanism that elucidates how gut microbes may influence GSD by regulating Omega-3 PUFA concentrations. This insight holds significant implications for informing future clinical practice. Nonetheless, this study has its limitations. Firstly, the majority of the GSD patients analyzed in the MR were of European origin, raising questions about the generalizability of these findings to the broader population. Secondly, there might be an overlap of participants between the exposure and outcome GWAS. Fortunately, the robust methods employed in this study, with a stringent F-statistic threshold (>10), help mitigate potential biases stemming from sample overlap (Burgess et al., 2016). Finally, despite the rigorous steps taken to identify and account for instrumental variable anomalies, the potential for horizontal pleiotropy effects may still be present. Moreover, it is crucial to acknowledge that MR analysis is a hypothesis-driven approach. Therefore, both experimental and clinical studies are imperative to establish the causal relationship between GM and GSD concerning the long-term effects of GM interventions.

## Conclusion

As far as we know, this is the initial study to thoroughly evaluate the causal connection between GM, bloodstream metabolites, and GSD. These results illuminate the potential mechanisms for clarifying the association between GM and GSD. Innovative concepts for microbiome- and metabolite-driven focused treatment and intervention in GSD are presented.

## Data availability statement

The original contributions presented in the study are included in the article/Supplementary material, further inquiries can be directed to the corresponding author.

## Author contributions

XH: Data curation, Investigation, Methodology, Software, Supervision, Writing – original draft, Writing – review & editing. QB: Investigation, Writing – original draft, Writing – review & editing. G-zS: Investigation, Software, Writing – original draft. YH: Software, Writing – review & editing. WQ: Data curation, Investigation, Methodology, Project administration, Software, Supervision, Writing – original draft, Writing – review & editing.

## References

[ref1] Arpaia N. Campbell C. Fan X. Dikiy S. van der Veeken J. deRoos P.. (2013). Metabolites produced by commensal bacteria promote peripheral regulatory T-cell generation. Nature 504, 451–455. doi: 10.1038/nature12726, PMID: 24226773 PMC3869884

[ref2] Aune D. Vatten L. J. Boffetta P. (2016). Tobacco smoking and the risk of gallbladder disease. Eur. J. Epidemiol. 31, 643–653. doi: 10.1007/s10654-016-0124-z, PMID: 26898907 PMC4977331

[ref3] Bodmer M. Brauchli Y. B. Krähenbühl S. Jick S. S. Meier C. R. (2009). Statin use and risk of gallstone disease followed by cholecystectomy. JAMA 302, 2001–2007. doi: 10.1001/jama.2009.1601, PMID: 19903921

[ref4] Burgess S. Davies N. M. Thompson S. G. (2016). Bias due to participant overlap in two-sample Mendelian randomization. Genet. Epidemiol. 40, 597–608. doi: 10.1002/gepi.21998, PMID: 27625185 PMC5082560

[ref5] Caruso R. Lo B. C. Núñez G. (2020). Host-microbiota interactions in inflammatory bowel disease. Nat. Rev. Immunol. 20, 411–426. doi: 10.1038/s41577-019-0268-7 32005980

[ref6] Cetta F. (1991). The role of bacteria in pigment gallstone disease. Ann. Surg. 213, 315–326. doi: 10.1097/00000658-199104000-00006, PMID: 2009013 PMC1358350

[ref7] Chen Y. Weng Z. Liu Q. Shao W. Guo W. Chen C.. (2019). FMO3 and its metabolite TMAO contribute to the formation of gallstones. Biochim. Biophys. Acta Mol. basis Dis. 1865, 2576–2585. doi: 10.1016/j.bbadis.2019.06.016, PMID: 31251986

[ref8] Diehl A. K. (2000). Cholelithiasis and the insulin resistance syndrome. Hepatology 31, 528–530. doi: 10.1002/hep.510310238 10655281

[ref9] Elsworth B. Lyon M. Alexander T. Liu Y. Matthews P. Hallett J.. The MRC IEU OpenGWAS data infrastructure. bio Rxiv (2020). 2020.08.10.244293.

[ref10] Grigor'eva I. N. Romanova T. I. (2020). Gallstone disease and microbiome. Microorganisms 8:6. doi: 10.3390/microorganisms8060835 PMC7356158 32498344

[ref11] Hu H. Shao W. Liu Q. Liu N. Wang Q. Xu J.. (2022). Gut microbiota promotes cholesterol gallstone formation by modulating bile acid composition and biliary cholesterol secretion. Nat. Commun. 13:252. doi: 10.1038/s41467-021-27758-8, PMID: 35017486 PMC8752841

[ref12] Islam K. B. Fukiya S. Hagio M. Fujii N. Ishizuka S. Ooka T.. (2011). Bile acid is a host factor that regulates the composition of the cecal microbiota in rats. Gastroenterology 141, 1773–1781. doi: 10.1053/j.gastro.2011.07.046, PMID: 21839040

[ref13] Jenkins T. C. Wallace R. J. Moate P. J. Mosley E. E. (2008). Board-invited review: recent advances in biohydrogenation of unsaturated fatty acids within the rumen microbial ecosystem. J. Anim. Sci. 86, 397–412. doi: 10.2527/jas.2007-0588, PMID: 18042812

[ref14] Kishino S. Takeuchi M. Park S. B. Hirata A. Kitamura N. Kunisawa J.. (2013). Polyunsaturated fatty acid saturation by gut lactic acid bacteria affecting host lipid composition. Proc. Natl. Acad. Sci. U. S. A. 110, 17808–17813. doi: 10.1073/pnas.1312937110, PMID: 24127592 PMC3816446

[ref15] Krumsiek J. Suhre K. Evans A. M. Mitchell M. W. Mohney R. P. Milburn M. V.. (2012). Mining the unknown: a systems approach to metabolite identification combining genetic and metabolic information. PLoS Genet. 8:e1003005. doi: 10.1371/journal.pgen.1003005, PMID: 23093944 PMC3475673

[ref16] Kurilshikov A. Medina-Gomez C. Bacigalupe R. Radjabzadeh D. Wang J. Demirkan A.. (2021). Large-scale association analyses identify host factors influencing human gut microbiome composition. Nat. Genet. 53, 156–165. doi: 10.1038/s41588-020-00763-1, PMID: 33462485 PMC8515199

[ref17] Kwek E. Zhu H. Ding H. He Z. Hao W. Liu J.. (2022). Peony seed oil decreases plasma cholesterol and favorably modulates gut microbiota in hypercholesterolemic hamsters. Eur. J. Nutr. 61, 2341–2356. doi: 10.1007/s00394-021-02785-9, PMID: 35107625

[ref18] Lammert F. Gurusamy K. Ko C. W. Miquel J. F. Méndez-Sánchez N. Portincasa P.. (2016). Gallstones. Nat. Rev. Dis. Primers. 2:16024. doi: 10.1038/nrdp.2016.24 27121416

[ref19] Leung J. W. Liu Y. L. Leung P. S. Chan R. C. Inciardi J. F. Cheng A. F. (2001). Expression of bacterial beta-glucuronidase in human bile: an *in vitro* study. Gastrointest. Endosc. 54, 346–350. doi: 10.1067/mge.2001.117546, PMID: 11522976

[ref20] Li G. Yu T. Du H. Zhang L. Liu X. Hou S. (2023). Effect of *Clostridium butyricum* on the formation of primary choledocholithiasis based on intestinal microbiome and metabolome analysis. J. Appl. Microbiol. 134, 1–10. doi: 10.1093/jambio/lxad170, PMID: 37533214

[ref21] Lin Z. Deng Y. Pan W. (2021). Combining the strengths of inverse-variance weighting and Egger regression in Mendelian randomization using a mixture of regressions model. PLoS Genet. 17:e1009922. doi: 10.1371/journal.pgen.1009922, PMID: 34793444 PMC8639093

[ref22] Liu X. Qi X. Han R. Mao T. Tian Z. (2023). Gut microbiota causally affects cholelithiasis: a two-sample Mendelian randomization study. Front. Cell. Infect. Microbiol. 13:1253447. doi: 10.3389/fcimb.2023.1253447, PMID: 37876873 PMC10591199

[ref23] Liu X. Tong X. Zou Y. Lin X. Zhao H. Tian L.. (2022). Mendelian randomization analyses support causal relationships between blood metabolites and the gut microbiome. Nat. Genet. 54, 52–61. doi: 10.1038/s41588-021-00968-y, PMID: 34980918

[ref24] Marschall H. U. Einarsson C. (2007). Gallstone disease. J. Intern. Med. 261, 529–542. doi: 10.1111/j.1365-2796.2007.01783.x 17547709

[ref25] Mathew L. K. Ko C. (2015). Dietary fat and protein intake are not associated with incident biliary sludge and stones during pregnancy. JPEN J. Parenter. Enteral Nutr. 39, 124–128. doi: 10.1177/0148607113520184, PMID: 24443325

[ref26] Molinero N. Ruiz L. Sánchez B. Margolles A. Delgado S. (2019). Intestinal Bacteria interplay with bile and cholesterol metabolism: implications on host physiology. Front. Physiol. 10:185. doi: 10.3389/fphys.2019.00185, PMID: 30923502 PMC6426790

[ref27] Nagpal R. Wang S. Ahmadi S. Hayes J. Gagliano J. Subashchandrabose S.. (2018). Human-origin probiotic cocktail increases short-chain fatty acid production via modulation of mice and human gut microbiome. Sci. Rep. 8:12649. doi: 10.1038/s41598-018-30114-4, PMID: 30139941 PMC6107516

[ref28] Petrov V. A. Fernández-Peralbo M. A. Derks R. Knyazeva E. M. Merzlikin N. V. Sazonov A. E.. (2020). Biliary microbiota and bile acid composition in Cholelithiasis. Biomed. Res. Int. 2020, 1–8. doi: 10.1155/2020/1242364 PMC7352139 32714973

[ref29] Ritchie S. C. Surendran P. Karthikeyan S. Lambert S. A. Bolton T. Pennells L.. (2023). Quality control and removal of technical variation of NMR metabolic biomarker data in ~120,000 UK biobank participants. Sci Data 10:64. doi: 10.1038/s41597-023-01949-y, PMID: 36720882 PMC9887579

[ref30] Sanderson E. (2021). Multivariable Mendelian randomization and mediation. Cold Spring Harb. Perspect. Med. 11:a038984. doi: 10.1101/cshperspect.a038984, PMID: 32341063 PMC7849347

[ref31] Sanna S. van Zuydam N. R. Mahajan A. Kurilshikov A. Vich Vila A. Võsa U.. (2019). Causal relationships among the gut microbiome, short-chain fatty acids and metabolic diseases. Nat. Genet. 51, 600–605. doi: 10.1038/s41588-019-0350-x, PMID: 30778224 PMC6441384

[ref32] Shama S. Liu W. (2020). Omega-3 fatty acids and gut microbiota: a reciprocal interaction in nonalcoholic fatty liver disease. Dig. Dis. Sci. 65, 906–910. doi: 10.1007/s10620-020-06117-5, PMID: 32036510 PMC7145364

[ref33] Takeda Y. Itoh H. Kobashi K. (1983). Effect of *Clostridium butyricum* on the formation and dissolution of gallstones in experimental cholesterol cholelithiasis. Life Sci. 32, 541–546. doi: 10.1016/0024-3205(83)90149-2, PMID: 6823210

[ref34] Trotman B. W. (1991). Pigment gallstone disease. Gastroenterol. Clin. N. Am. 20, 111–126. doi: 10.1016/S0889-8553(21)00536-7 2022417

[ref35] Tsai C. J. Leitzmann M. F. Willett W. C. Giovannucci E. L. (2004). The effect of long-term intake of cis unsaturated fats on the risk for gallstone disease in men: a prospective cohort study. Ann. Intern. Med. 141, 514–522. doi: 10.7326/0003-4819-141-7-200410050-00007, PMID: 15466768

[ref36] Tsai C. J. Leitzmann M. F. Willett W. C. Giovannucci E. L. (2006). Central adiposity, regional fat distribution, and the risk of cholecystectomy in women. Gut 55, 708–714. doi: 10.1136/gut.2005.076133, PMID: 16478796 PMC1856127

[ref37] Wang J. Zhang H. Chen X. Chen Y. Menghebilige B. Q. (2012). Selection of potential probiotic lactobacilli for cholesterol-lowering properties and their effect on cholesterol metabolism in rats fed a high-lipid diet. J. Dairy Sci. 95, 1645–1654. doi: 10.3168/jds.2011-4768, PMID: 22459813

[ref38] Wu T. Zhang Z. Liu B. Hou D. Liang Y. Zhang J.. (2013). Gut microbiota dysbiosis and bacterial community assembly associated with cholesterol gallstones in large-scale study. BMC Genomics 14:669. doi: 10.1186/1471-2164-14-669, PMID: 24083370 PMC3851472

[ref39] Xu H. Jurado-Fasoli L. Ortiz-Alvarez L. Osuna-Prieto F. J. Kohler I. Di X.. (2022). Plasma levels of Omega-3 and Omega-6 derived Oxylipins are associated with fecal microbiota composition in young adults. Nutrients 14:4991. doi: 10.3390/nu14234991, PMID: 36501021 PMC9736377

[ref40] Yao Y. Cai X. Fei W. Ye Y. Zhao M. Zheng C. (2022). The role of short-chain fatty acids in immunity, inflammation and metabolism. Crit. Rev. Food Sci. Nutr. 62, 1–12. doi: 10.1080/10408398.2020.1854675 33261516

[ref41] Ye X. Huang D. Dong Z. Wang X. Ning M. Xia J.. (2022). FXR signaling-mediated bile acid metabolism is critical for alleviation of cholesterol gallstones by Lactobacillus strains. Microbiol. Spectr. 10:e0051822. doi: 10.1128/spectrum.00518-22, PMID: 36036629 PMC9603329

[ref42] Ye X. Shen S. Xu Z. Zhuang Q. Xu J. Wang J.. (2021). Sodium butyrate alleviates cholesterol gallstones by regulating bile acid metabolism. Eur. J. Pharmacol. 908:174341. doi: 10.1016/j.ejphar.2021.174341, PMID: 34273384

[ref43] Zhang Z. Cheng L. Ning D. (2023). Gut microbiota and sepsis: bidirectional Mendelian study and mediation analysis. Front. Immunol. 14:1234924. doi: 10.3389/fimmu.2023.1234924, PMID: 37662942 PMC10470830

